# Definition and Independent Validation of a Proteomic-Classifier in Ovarian Cancer

**DOI:** 10.3390/cancers12092519

**Published:** 2020-09-04

**Authors:** Sabine Kasimir-Bauer, Joanna Roder, Eva Obermayr, Sven Mahner, Ignace Vergote, Liselore Loverix, Elena Braicu, Jalid Sehouli, Nicole Concin, Rainer Kimmig, Lelia Net, Heinrich Roder, Robert Zeillinger, Stefanie Aust, on behalf of the OVCAD (Ovarian Cancer Diagnosis initiative)

**Affiliations:** 1 Department of Gynecology and Obstetrics, University Hospital of Essen, Hufelandstr. 55, 45147 Essen, Germany; Sabine.Kasimir-bauer@uk-essen.de (S.K.-B.); rainer.kimmig@uk-essen.de (R.K.); 2 Biodesix, 2970 Wilderness Place #100, Boulder, CO 80301, USA; joanna.roder@biodesix.com (J.R.); lelia.net@biodesix.com (L.N.); heinrich.roder@biodesix.com (H.R.); 3 Department of Obstetrics and Gynecology, Medical University of Vienna, Molecular Oncology Group, Gynecologic Cancer Unit, Comprehensive Cancer Center, 1090 Vienna, Austria; eva.obermayr@meduniwien.ac.at (E.O.); stefanie.aust@meduniwien.ac.at (S.A.); 4 Department of Gynecology, University Medical Center Hamburg-Eppendorf, 20251 Hamburg, Germany; s.mahner@uke.de; 5 Department of Gynecologic Oncology, Leuven Cancer Institute, University Hospitals Leuven, KU Leuven, B-3000 Leuven, Belgium; ignace.vergote@uzleuven.be (I.V.); liselore.loverix@kuleuven.be (L.L.); 6 Department of Gynecology, Charité University Medicine, Campus Virchow, 13353 Berlin, Germany; elena.braicu@charite.de (E.B.); Jalid.Sehouli@charite.de (J.S.); 7 Department of Gynecology and Obstetrics, Innsbruck Medical University, 6020 Innsbruck, Austria; nicole.concin@i-med.ac.at

**Keywords:** ovarian cancer, proteomics, mass spectrometry, survival

## Abstract

**Simple Summary:**

The heterogeneity of epithelial ovarian cancer and its associated molecular biological characteristics are continuously integrated in the development of therapy guidelines. In a next step, future therapy recommendations might also be able to focus on the patient’s systemic status, not only the tumor’s molecular pattern. Therefore, new methods to identify and validate host-related biomarkers need to be established. Using mass spectrometry, we developed and independently validated a blood-based proteomic classifier, stratifying epithelial ovarian cancer patients into good and poor survival groups. We also determined an age dependence of the prognostic performance of this classifier and its association with important biological processes. This work highlights that, just like molecular markers of the tumor itself, the systemic condition of a patient (partly reflected in proteomic patterns) also influences survival and therapy response and could therefore be integrated into future processes of therapy planning.

**Abstract:**

Mass-spectrometry-based analyses have identified a variety of candidate protein biomarkers that might be crucial for epithelial ovarian cancer (EOC) development and therapy response. Comprehensive validation studies of the biological and clinical implications of proteomics are needed to advance them toward clinical use. Using the Deep MALDI method of mass spectrometry, we developed and independently validated (development cohort: *n* = 199, validation cohort: *n* = 135) a blood-based proteomic classifier, stratifying EOC patients into good and poor survival groups. We also determined an age dependency of the prognostic performance of this classifier, and our protein set enrichment analysis showed that the good and poor proteomic phenotypes were associated with, respectively, lower and higher levels of complement activation, inflammatory response, and acute phase reactants. This work highlights that, just like molecular markers of the tumor itself, the systemic condition of a patient (partly reflected in proteomic patterns) also influences survival and therapy response in a subset of ovarian cancer patients and could therefore be integrated into future processes of therapy planning.

## 1. Introduction

Despite all the advances in ovarian cancer research, and the understanding of the heterogeneity of this disease, epithelial ovarian cancer (EOC) still has the highest mortality rate of all gynecological malignancies, with a five-year survival in advanced stages of only 30%. Primary therapy for patients with EOC is radical cytoreductive surgery, followed by adjuvant platinum-based chemotherapy, administered in six cycles of carboplatin and paclitaxel with/without bevacizumab [1]. More recently, PARP (Poly(ADP-ribose)-Polymerase)-inhibition was also introduced in the first-line-treatment for selected patients [2]. Initial response to therapy can be divided into three categories: platinum-resistant or -refractory, platinum-partially sensitive, and platinum-responsive. The duration of response to primary chemotherapy is considered the most important factor for treatment of recurrence [3]. Management of EOC could be improved by the use of validated biomarkers to identify patients, in advance of therapy, that are likely to be platinum resistant and at high risk of early progression [4].

The potential of proteomics-guided therapy stratification in EOC was highlighted over a decade ago [5,6]. Mass spectrometry (MS) is one of the main technologies used in protein biomarker development, producing reproducible measurements [7]. Based on plasma or serum samples, this minimally invasive technique can be routinely performed in a clinical laboratory. MS-based analyses have identified a variety of candidate protein biomarkers that might be crucial for EOC development and diagnosis [8,9,10], and the FDA-cleared Ova1 test (Vermillion, Austin, TX, USA) is based on differentially expressed proteins originally identified by using a Surface-Enhanced Laser Desorption/Ionization (SELDI)–time of flight (TOF) MS platform [11,12,13].

To address the unmet need for non-invasive tests to select patients likely not to respond to standard treatment in EOC [5], we here describe the development and independent validation of an MS-based plasma proteomic classifier that stratifies patients with primary EOC prior to standard treatment. As standardization and optimization of protocols are of utmost importance in cancer research, we made use of a Matrix-Assisted Laser Desorption/Ionization (MALDI)–TOF platform and the Deep MALDI method of MS, extending the observable dynamic range in a single workflow [14]. These processed spectral data were combined with clinical outcome data, using a machine learning approach optimized for the personalized medicine setting, where there are typically more measured attributes than patient samples [15,16]. The resulting test was completely locked before application to the independent validation cohort, assessment of underlying biology, and evaluation of test reproducibility.

## 2. Results

### 2.1. Patients and Samples

Plasma samples obtained within one week before surgery were used for classifier development and validation. The development cohort (DC) comprised 199 patients with EOC treated at the Department of Gynecology and Obstetrics at University of Essen, Germany, diagnosed between 2004 and 2014. Median overall survival (OS) of the DC was 63 months (95% Confidence Interval (CI) 47 months, undefined). The independent validation cohort (VC) comprised 135 patients with EOC treated within the European OVCAD consortium (Berlin, Hamburg (Germany), Leuven (Belgium) and Innsbruck, Vienna (Austria)), diagnosed between 2005 and 2008 [17]. Median OS of the VC was 52 months (95% CI: 43–64 months). All patients had histologically confirmed EOC. Patient characteristics are shown in Table 1. All patients were treated according to the guidelines that were recommended during the indicated study period, namely radical cytoreductive surgery and platinum-based chemotherapy. Median follow-up for both cohorts exceeded five years. A total of 87 DC and 76 VC patients died within the follow-up period of the respective studies.

As we had hypothesized that outcome-related molecular signatures could differ between younger and older patients, for test development and performance assessment, DC patients were divided into two subgroups, by age: age 55 or younger (77 patients (39%)) and age 56 or older (122 patients (61%)). The threshold of 55 years of age was chosen to be close to menopause and to allow enough patients in each subgroup for analysis.

### 2.2. Development Cohort

Data from each age category (≤55 years and ≥56 years) were used separately to generate two tests (classifiers) able to stratify patients into two subgroups: “good” and “poor” with better and worse outcomes, respectively. The tests were then applied to the mass spectral data of all patients in the DC. Out-of-bag methods [18] were used to produce reliable test classifications for patients whose data were used in classifier creation. Performance of the two tests, trained using data from older and younger patients, respectively, were quite similar (see Figure S1), and we decided to continue using only the test trained using data from the older patients. All subsequent results presented herein pertain to the test trained on the older patient subgroup from the DC.

**Figure 1:**
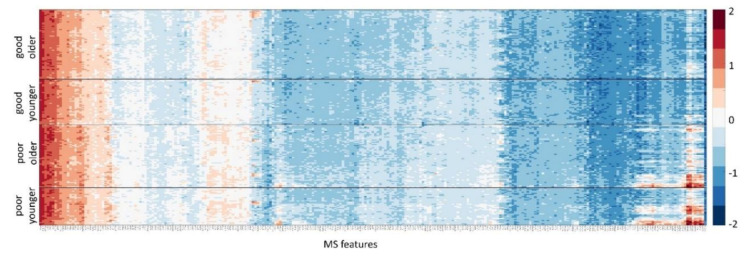
Heatmap of the natural logarithm of features used in the test. Samples in the development cohort are shown as rows, grouped by test classification (good or poor) and age category (younger (age ≤ 55) and older (age ≥ 56)). Mass spectral features are shown as columns. Features and samples are hierarchically clustered.

A heatmap of the 269 mass-spectral features used in the test for each of the samples in the DC is shown in Figure 1. Results of the analysis of univariate association of each MS feature with test classification are contained in the Supplementary Materials.

Of the 199 patients in the DC, 106 (53%) were assigned to the good group, and the remaining 93 (47%) to the poor group. Within younger patients (*n* = 77), 55% (42/77) were classified as good; in the subgroup of older patients, 52% (64/122) were classified as good. Patient characteristics of each subgroup are summarized in the Supplementary Materials overall and by test classification (Tables S1–S3)**.** Test classification was associated with known baseline prognostic factors, including histology, grade, and stage (Table S1). In addition, classification was strongly associated with presence of residual tumor after surgery. Seventy-four percent (79/106) of patients classified as good had no tumor remaining after surgery, compared with only 38% (35/93) of patients classified as poor (Fisher’s exact test *p* < 0.001). This correlation was particularly clear in patients age 55 or younger, where 86% (36/42) of the good group had no residual tumor after surgery, compared with only 37% (13/35) of patients in the poor group (*p* <0.001).

#### Survival Analyses

**Figure 2:**
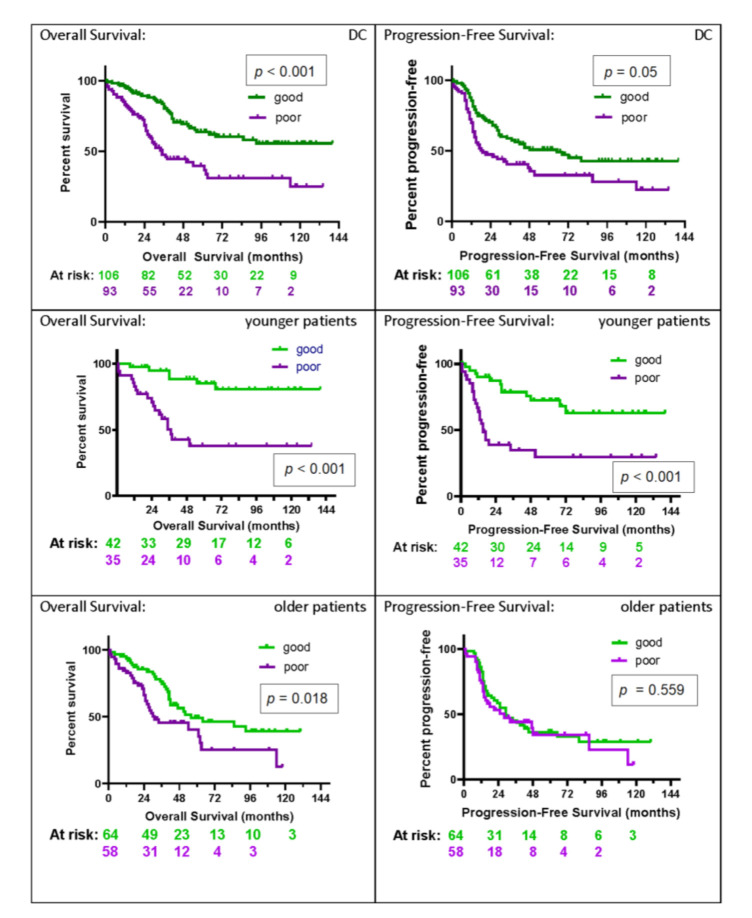
Kaplan–Meier (KM) plots by test classification (good vs. poor) of overall and progression-free survival for the development cohort (DC): overall (DC, *n* = 199) and for younger patients (age ≤ 55, *n* = 77) and older patients (age ≥ 56, *n* = 122) separately. The number of patients at risk in the KM analysis at two-year intervals in each test classification group (green = good, and purple = poor) is shown below the *x*-axis.

Kaplan–Meier (KM) plots for OS and progression-free survival (PFS) for the whole DC, as well as the subgroups of younger (age ≤ 55) and older patients (age ≥ 56), are shown in Figure 2.

In younger patients, both OS and PFS show significant differences between test classification groups (*p* < 0.001 and *p* < 0.001, respectively), with substantial effect sizes for each (hazard ratios (HRs) OS: HR 0.18 (95% CI: 0.07–0.45); PFS: HR 0.28 (95% CI: 0.14–0.58)). One-, two-, and five-year survival rates were 83%, 71%, and 38% for the poor group and 98%, 95%, and 85% for the good group. Median PFS was 15 (95% CI: 11–51) months for the poor group, whereas, in the good group, the median was not reached. PFS rates at six months and 12 months were 85% and 64%, respectively, for the poor group, and 95% and 90%, respectively, for the good group.

In the subgroup of older patients, patients classified as good had significantly better OS (HR = 0.55 (95% CI: 0.33–0.90); *p* = 0.018). Median OS was 56 (95% CI: 41-undefined) months for patients classified as good and 31 (95% CI: 25–62) months for patients classified as poor. One-, two-, and five-year survival rates were 83%, 66%, and 40% for the poor group and 94%, 85%, and 49% for the good group. No significant differences were seen in PFS (*p* = 0.559).

**Figure 3:**
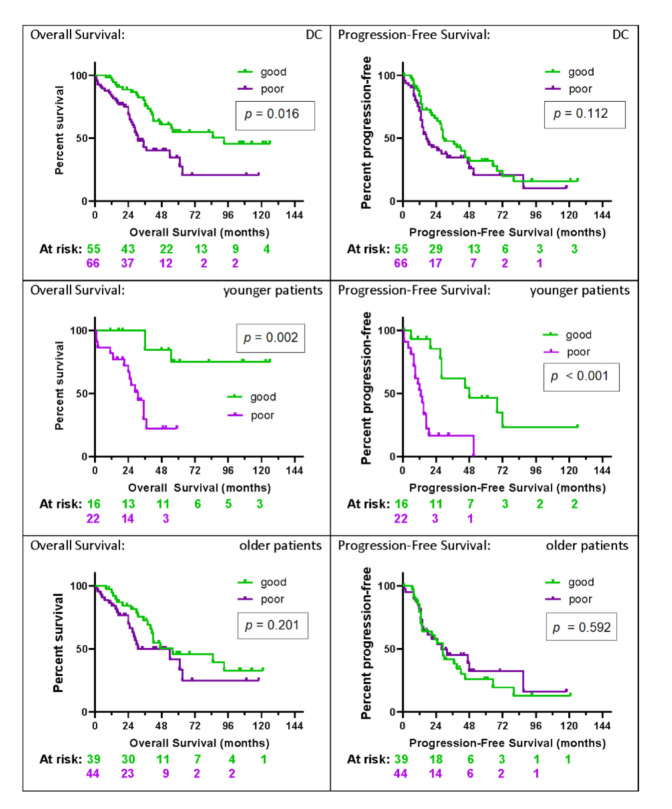
KM plots by test classification of overall and progression-free survival for all patients (*n* = 121), younger patients (*n* = 38), and older patients (*n* = 83) with advanced-stage high-grade serous ovarian carcinoma in the development cohort. The number of patients at risk in the KM analysis at two-year intervals in each test classification group (green = good, and purple = poor) is shown below the *x*-axis.

As a test predictive of outcome would be of most potential utility for patients with advanced, high-grade serous EOC, a subgroup analysis was performed in patients with serous histology, FIGO statuses III and IV, and tumor grades 2 and 3 (*n* = 121; Figure 3). Fifty-five (45%) of the 121 patients with high-grade serous advanced-stage disease were assigned a good test classification, whereas 66/121 (55%) were identified as poor. Within younger patients, 16/38 (42%) were classified as good, and in older patients, 39/83 (47%) were classified as good. As is apparent from Figure 3, significant differences were obtained between the good and poor subgroups within younger patients for OS (HR = 0.13 (95% CI: 0.03–0.46), *p* = 0.002) and PFS (HR = 0.19 (95% CI: 0.08–0.48), *p* < 0.001). Median OS and PFS were only 31 months and 13 months, respectively, for younger patients classified as poor. One-, two-, and five-year survival was 100%, 100%, and 75% for younger patients classified as good, and 82%, 67%, and 22% for those classified as poor. Six- and twelve-month PFS was 93% and 93% vs. 81% and 53% for good vs. poor subgroups of younger patients, respectively. The test was not able to provide a meaningful stratification of outcomes for older patients with high-grade advanced-stage serous EOC (*p* = 0.201 for OS and *p* = 0.592 for PFS).

### 2.3. Validation Cohort

Within the independent VC (*n* = 135), the test classified 66 patients (49%) to the good group and the remaining 69 patients (51%) to the poor group. In the subgroups of younger/older patients (age ≤ 55, *n* = 58; age ≥ 56 years, *n* = 77), 43%/53% were classified as good, respectively. Test classification was associated with grade (*p* = 0.049; see Table S4). Test classification was not associated with presence of tumor after surgery in the VC (*p* = 0.345). However, 92% of the younger patients (23/25, age ≤ 55) classified as good had no residual tumor post-surgery, compared to 73% (24/33) of patients classified as poor (Fisher exact test *p* = 0.093).

#### Survival Analyses

**Figure 4:**
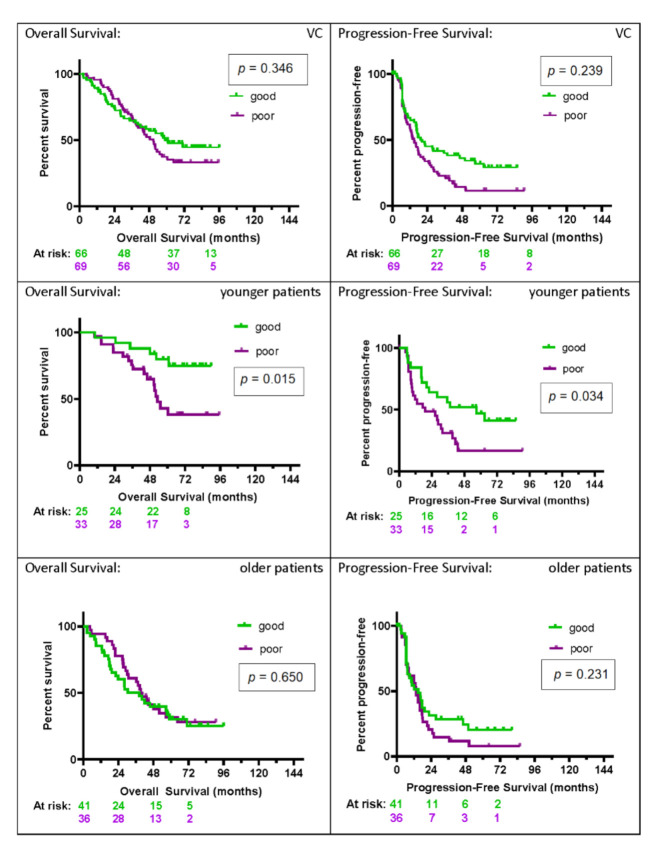
KM plots by test classification (good vs. poor) of overall and progression-free survival for the validation cohort (VC): overall (VC, *n* = 135) and for younger patients (age ≤ 55, *n* = 58) and older patients (age ≥ 56, *n* = 77) separately. The number of patients at risk in the KM analysis at two-year intervals in each test classification group (green = good, and purple = poor) is shown below the *x*-axis.

Kaplan–Meier plots for OS and PFS for the VC, as well as the subgroups of younger and older patients, are shown in Figure 4. Younger patients (age ≤ 55; *n* = 58) had significantly longer OS and PFS if classified as good (*p* = 0.015 and *p* = 0.034, respectively), with substantial effect sizes for each (hazard ratios OS: HR 0.31 (95% CI: 0.12–0.79); PFS: HR 0.48 (95% CI: 0.25–0.95)). In contrast to the poor group, with one-, two-, and five-year survival rates of 97%, 85%, and 43% and a median OS time of 53 (95% CI: 44-undefined) months, the values for the good group were 96%, 96%, and 80%, with the median OS not reached. Median PFS was 19 (95% CI: 9–32) months and 57 (95% CI: 20-undefined) months in the poor and good group, respectively. PFS rates at six and 12 months were 94% and 58%, respectively, for the poor group, and 96% and 84%, respectively, for the good group.

Comparing good versus poor in the group of older patients (age ≥ 56; *n* = 77), we see that the test was unable to stratify PFS or OS. OS was similar for good and poor groups (*p* = 0.650). Median survival was 40 (95% CI: 29–57) months for the poor group and 38 (95% CI: 19–57) months for the good group. No significant differences were seen in PFS, with a median PFS of 13 (95% CI: 8–17) months in the poor group and 15 (95% CI: 7–19) months in the good group.

### 2.4. Multivariate Analyses DC and VC

In the younger patient subgroup of the DC, the classification was a clear univariate predictor of both OS and PFS and remained a significant predictor of OS and PFS in multivariate analysis adjusted for known prognostic factors (Table 2). Hence, although test classification was associated with many prognostic factors (see Supplementary Materials), it provided additional information on outcome, complementary to these other factors. Separate analyses adjusting separately for FIGO stage and residual tumor alone are contained within Tables S5 and S6 and support this conclusion.

In the VC younger patient subgroup, the point values of hazard ratios were not appreciably diminished when other important prognostic factors were included in the multivariate analysis; however, the associated *p*-values increased slightly above the 0.05 level, likely due to small sample size (*n* = 58).

### 2.5. Detailed Analysis of Age Dependence

A more detailed analysis in the DC of age dependence of the ability of the test to stratify patient outcome showed evidence of a steady deterioration with increasing age, with no evidence of stratification power for the oldest patients. In particular, it was noted that the dependence of outcome on age and test classification could be well captured in a product of the two attributes (ProteomicAgeClassification = 0 for patients classified as poor, and ProteomicAgeClassification = 0.1 (80-Age in years) for patients classified as good). The results of multivariate analysis of OS and PFS, using this compound variable, are given in Table 3. Multivariate analysis in the validation set verified the significance of this compound variable as an independent predictor of OS and PFS. Note that a ProteomicAgeClassification HR of 0.80 indicates that outcomes deteriorate by 20% for each ten-year age increase for patients with a good classification, while patients classified as poor have similar outcomes regardless of age.

### 2.6. Test Reproducibility

Previous studies have examined in detail the analytical reproducibility of Deep MALDI mass spectra [14]. Using the same mass spectrometer and spectral acquisition procedures, it was shown, for a similar set of 298 mass spectral features as used in this work, that the median CV across features was 2.3% with interquartile range 1.4–5.0%. Here, we focus on the impact of variation in the acquired mass spectra on the reproducibility of the test classification. Test classification reproducibility was assessed by rerunning the test on a subset of 34 samples from the younger patients in the DC. The entire test procedure, from thawing samples, preparing samples, acquiring mass spectra, processing mass spectra to classifying the processed spectra data, was repeated. Concordance of test classifications between the two runs was 97% (see Table S7). A subset of the younger patients (*n* = 37) from the DC had serum and plasma samples collected at the same time. Analysis of these serum samples by identical procedures showed a concordance between test classifications generated from serum and plasma of 89% (see Table S8), indicating the likelihood that the test could be run on serum as well as plasma samples.

### 2.7. Protein Set Enrichment Analysis (PSEA)

The association of various biological processes with test classifications was investigated by using PSEA methods. Set enrichment analysis was carried out by using the Reference Sample Set (*n* = 46), for which both protein panel expression and mass spectral data were available. A test classification was generated for each sample in the Reference Sample Set. Twenty samples (43%) were classified as poor, and the remaining 26 samples (57%) were classified as good. Univariate correlation of protein expression from the SOMAscan assay with test classification with Mann–Whitney *p* < 0.001 and false discovery rates (FDRs) < 0.10 were found for the following proteins: complement C2, complement factor B, retinol-binding protein 4, kallistatin, complement protein C9, C reactive protein, insulin-like growth factor-binding protein 1, and D-dimer. (Results for all 1305 proteins in the panel and the membership of proteins within the subsets associated with each biological process investigated are provided in the Table S9.)

Set enrichment methods were used to determine the association between biological processes of interest and test classification. The good test classification was associated with lower levels of complement activation (*p* = 0.002), acute inflammation (*p* = 0.003), and acute phase reactants (*p* = 0.001), all with false discovery rates of <0.03. A table showing the full set enrichment analysis results is in Table S10.

## 3. Discussion

In the era of increasing affordability and applicability of proteomic analyses, the treatment of a variety of cancers has been diversified. Novel non-invasive platforms, including proteomics of blood-based samples, have evolved during recent years [19]. In this study, we identified a proteomic classifier stratifying patients into a “good” phenotype and “poor” phenotype, with respectively better and worse OS and PFS.

The classifier was first developed in a development cohort of 199 EOC patients. A significant association between PFS or OS and the stratification according to the proteomic classifier was then seen only in patients younger than 56 years of age. PFS rates at 12 months were 64% for the poor-signature and 90% for the good-signature group. This effect translated to OS, with a five-year survival rate of 38% compared to 85% for the poor-signature and good-signature group, respectively. The test was also able to provide a meaningful stratification of outcomes in the subgroup of younger patients with advanced-stage high-grade serous ovarian cancer. The classifier thus helps to identify a subgroup of patients likely to respond less well to standard therapy. Current standard treatment of EOC consists of surgery, followed by adjuvant platinum-based chemotherapy, with/without bevacizumab or PARP-inhibitors as maintenance therapy. Since chemotherapy-resistance is a major problem in EOC, selection criteria are needed to identify patients in need of alternative therapies. Unfortunately, treatment options for EOC patients not likely to respond to platinum-based chemotherapy are limited. Still, dose-dense protocols could be proposed [20] for patients at highest risk of recurrence or progression, and acceleration of molecular analyses for precision tumor boards to determine actionable molecular alterations [21], followed by advanced access to targeted therapies, could be discussed.

We saw good performance of the developed classifier in younger patients (≤55 years) not only in the DC, but also in an independent VC. A significant association between PFS or OS and the stratification, according to the proteomic classifier, was seen in the younger patient subgroup of the VC.

As performance of the classifier demonstrated a consistent dependence on age in both the DC and the VC, we analyzed the age dependence of the classifier and found a deterioration with increasing age, with no evidence of proteomic-based stratification power for the oldest patients. A newly defined variable combining age and the proteomic classification showed significance, independent of known prognostic parameters for OS and PFS in the DC, and we verified the significance of this compound variable as an independent predictor of both OS and PFS in the VC. An age-dependence of proteomic measurements is not surprising, as aging of different cell types and tissues results in proteomic changes [22]. Lehallier et al. described a significant overlap between disease proteomes and the waves of aging proteins [23], and proteomics is frequently used in aging research [24,25]. However, it is of note that our observations follow from age-dependent changes in the association of our proteomic phenotypes with outcome, rather than from our measurement of age dependent changes in the proteome itself. Our data indicate that the significance of proteomics-based research in EOC is increased if age is accounted for in the analyses.

The test classification was strongly associated with the presence of residual tumor after surgery in the DC (*p* < 0.001). Although this association did not reach the level of statistical significance in the VC, it would be interesting to further investigate the impact of MS-based algorithms, together with previously described criteria [26,27], for predicting incomplete cytoreduction in advanced EOC.

The aim of this analysis was to identify EOC patients in need of alternative treatment approaches (poor responders to standard treatment) by focusing particularly on patients’ proteomic-systemic condition. A similar strategy has been successfully investigated in lung cancer patients, where an MS-based proteomic biomarker classification (the VeriStrat^®^ test; Biodesix, Boulder, CO, USA) was first developed in training and validation sets [28] and further tested and validated in a variety of prospective and retrospective clinical trials: The predictive and prognostic effects on response and survival were validated in a subset of patients enrolled in the NCIC Clinical Trials Group, BR.21 phase III trial of erlotinib versus placebo in previously treated advanced non-small-cell lung cancer patients [29]. In a prospective phase III trial designed to investigate the predictive potential of the biomarker between second-line erlotinib or chemotherapy in non-small-cell lung cancer, patients were stratified into a “good signature” group and a “poor signature” group. Patients with a poor VeriStrat proteomic test classification had worse survival with erlotinib than chemotherapy (HR 1.72; 95% CI, 1.08–2.74; *p* = 0.022) [30], and the test was predictive of differential benefit between the two therapies. Furthermore, pretreatment VeriStrat status was correlated with survival in the phase III LUX-Lung 8 study of 795 patients [31]. Further studies have indicated that the MS-based proteomic classifier is likely detecting a tumor–host response to the presence of the cancer [29,32].

Why does the proteome matter? Not every patient has the same systemic condition. As we have started to better understand that tumor heterogeneity entails that different treatment strategies need to be defined according to the tumor’s molecular biology, there is also a need to incorporate the patients’ (hosts’) individual variability in treatment planning. In the light of an increasing number of immunotherapy trials in EOC [33] (e.g., ClinicalTrials.gov Identifier: NCT02891824, NCT02718417, NCT02674061, NCT02498600, NCT02484404, and NCT03737643), it becomes more and more evident that the “heterogeneity” of the patient’s systemic condition, such as the individual immunological host-response to the cancer, also needs to be taken into account when establishing the optimal treatment for our patients. Beyond cell-free DNA and circulating tumor DNA originating from normal and cancer cells, soluble protein and peptides represent biomolecular analytes that can easily be measured for clinical diagnostic purposes.

Here we used the Deep MALDI method of mass spectrometry, resulting in a deeper probing of the proteome, by increasing the signal-to-noise ratio of the measurements and an increased number of measurable circulating proteins from human blood samples [14]. This technique has recently been used for outcome stratification of patients receiving immunotherapy [34,35,36].

Protein set enrichment analysis indicated that test classification was associated with complement activation, acute phase reactants, and acute inflammation. Test classification was also shown to be associated with expression of complement C2, complement factor B, RBP4, kallistatin, C9, CRP, insulin-like growth factor-binding protein 1 (IGF-1), and D-dimer. Association of these proteins with prognosis in cancer, in general, and EOC, in particular, has been observed in previous studies [37,38,39,40,41,42,43]. In this regard, meta-analyses demonstrated that high CRP levels were associated with increased risk of invasive EOC [38], whereas no significant association of IGF-1/IGFBP-3 with EOC risk was identified [39]. In addition, RBP4 was shown to drive ovarian cancer cell migration [40]; pretreatment plasma D-dimer levels were associated with chemoresistance and poor disease outcome [41,42]; and low expression of kallistatin was associated with unfavorable prognosis, platinum resistance, and relapse [43]. These results indicate a potential relation to the individual immunological host-response to cancer. Additionally, systemic inflammatory response is known to be associated with cancer cachexia and thus muscle atrophy, weakness, fatigue, and reduced survival in patients with advanced EOC [44]. Patients with peritoneal metastasis often present with a nutritional deficit and cancer cachexia [45]. The proteomic signature of these patients might be of particular importance in treatment planning.

The use of multisite samples in the VC derived from the EU-project OVCAD [17,46] allowed us to test that our results are “robust” against slight differences that might occur between sites in sample collection and storage conditions [13]. Additionally, as reproducibility is essential in cancer research, we reran the test from scratch (including sample preparation, and spectral acquisition and processing and classification) in a subset of patients, reaching an agreement of 97% in classification accuracy. Concordance of 89% was seen between test classifications generated from serum and plasma samples obtained simultaneously from a subset of patients in this study, indicating that the proposed procedure might be feasible in both serum and plasma samples. As a patient’s proteomic signature reflects a specific health condition at a given time point, it may be essential for therapy guidance [47]. We used mass-spectral data and machine learning to develop a classifier that stratifies patients into two proteomic phenotypes. Patients in the “good” phenotype exhibited worse outcomes with increasing age, whereas patients in the “poor” phenotype had poor outcomes regardless of age. For younger patients (<56 years of age), patients classified as “good” had significantly better outcomes than those classified as “poor”. The association of the proteomic classifications with biological processes such as acute inflammation indicates a potential relation to the individual immunological host-response to cancer. We believe that combining molecular characteristics of the tumor and biomarkers of the hosts’ systemic condition might enhance therapy selection in the future.

## 4. Material and Methods

### 4.1. Cohorts and Patient Characteristics

Written informed consent was obtained from all patients, and the study was approved by the respective local ethics committees and performed according to the Declaration of Helsinki (ethics code: 05-2870 (Essen), EK366/EK260 (Vienna), EK207/2003 (Berlin), ML2524 (Leuven), HEK190504 (Hamburg)). The data processing was completely anonymized. Tumors were classified according to the World Health Organization classification of tumors of the female genital tract. Grading was conducted by using the grading system proposed by Silverberg, and tumor staging was classified according to the Fédération Internationale de Gynécology et d’Obstétrique (FIGO).

In addition to the development and validation cohort already described, an independent set of 46 serum samples collected from 46 female patients with cancer (Reference Sample Set) was used for set enrichment analyses of the association of various biological processes with classification groups of the developed test. These samples were obtained from commercial biobanks (Conversant Bio (Huntsville, AL, USA) and Oncology Metrics (Forth Worth, TX, USA)), under their ethics-approved protocols.

### 4.2. Spectral Acquisition

**Figure 5:**
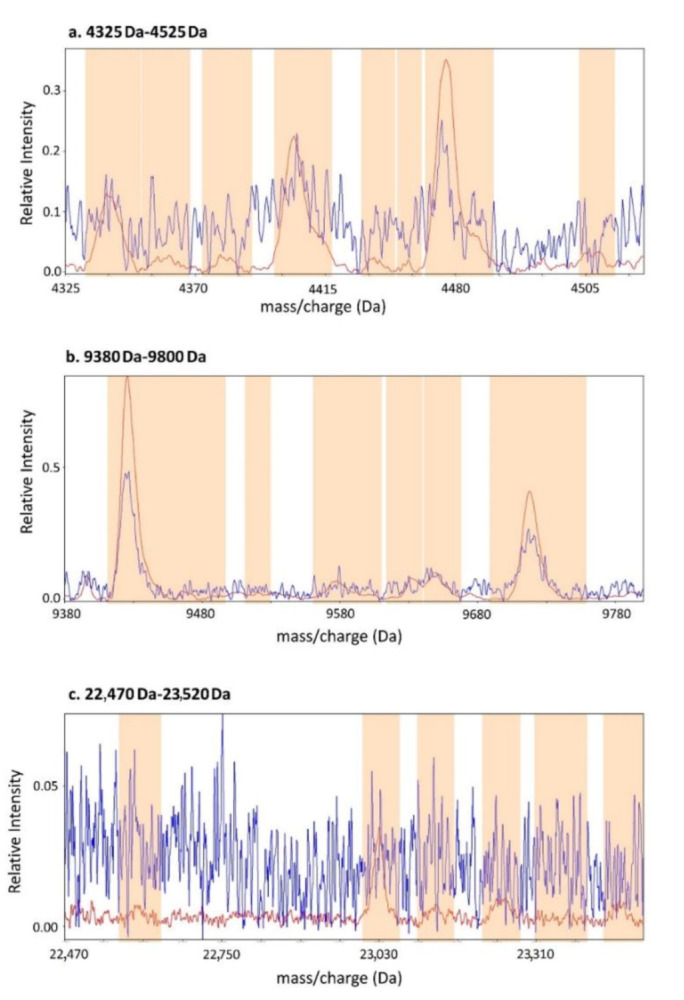
Comparison of a conventional MALDI spectrum with a Deep MALDI spectrum. A 2400-shot conventional spectrum (blue) and a 400,000-shot Deep MALDI spectrum (red) for a sample in the development cohort are shown for three regions of the mass spectrum: (**a**) 4325–4535 Da, (**b**) 9380–9800 Da, and (**c**) 22,470–23,520 Da. The orange bands show the locations of mass-spectral features.

Spectral acquisition and processing were performed by using the Deep MALDI method of mass spectrometry on a SimulToF mass spectrometer (SimulTof Systems, Marlborough, MA, USA). This approach, requiring only 3 µL of plasma, allows a for deeper probing of the proteome (i.e., assessment of peaks spanning a higher range of intensities) than is possible with conventional MALDI methods, by exposing the samples to many more laser “shots” (400,000, as compared with typically 1000–10,000) [14] (see Figure 5).

The resulting spectra were processed to render them reproducible and comparable between samples, as has been described elsewhere [34] (see also Supplementary Materials including Tables S10–S14). Mass spectral features were defined as regions in the mass spectra containing identifiable peaks in at least some spectra, see Figure 5 and Figure S2. Features known to demonstrate poor reproducibility or related to sample hemolysis were not used, leaving 269 mass spectral features (listed in the Supplementary Materials, Table S15) for analysis. All parameters were defined by using only the development set of samples, and this fully fixed procedure was applied to the validation cohort without modification.

### 4.3. Test Development

Spectral data from the development set of samples were used to generate a test able to stratify patients into two groups, good and poor, with better and worse prognosis, respectively. All 269 mass-spectral features were used without any feature selection or deselection based on clinical data. Test generation was carried out by using an approach which incorporates concepts from traditional machine learning and deep learning and is designed for test development in cases with more measured attributes than samples, minimizing the potential for overfitting and promoting the ability of the resulting test to generalize to unseen datasets [15] (see Supplementary Materials including Figure S3). This method splits the development cohort into training and test sets multiple times, generates a classifier for each training set, and averages over the ensemble of training/test set splits. Reliable performance estimates for the development cohort can therefore be obtained by classifying each sample by using data generated only when it is in a test set and not used in training.

The classification algorithm within each training set split is constructed from the mass-spectral features as a combination of many k-nearest neighbor classifiers (k = 9), which individually have at least minimal power to stratify patients by overall survival. This combination is strongly regularized to prevent overfitting to specific details of the training set. A semi-supervised approach, which allows simultaneous refinement of the test and the classes used in its training, reveals the underlying structure of the mass spectral data associated with the outcome [16], Figure S4. Full details of classifier training are provided in the Supplementary Materials. This method of classifier development has been used previously with Deep MALDI mass spectral data to create validated tests able to stratify outcomes for melanoma patients treated with immunotherapy [34,35]. The parameters and all reference data for the final classifier were generated solely on the development cohort and were then locked. Validation was performed by using this classifier in the VC.

### 4.4. Protein Set Enrichment Analysis (PSEA)

Gene set enrichment analysis methods were applied to protein expression data for the Reference Sample Set, for which mass spectral data were also available, to investigate the biological underpinnings of the test classifications [48]. Test classifications were generated for each sample in the Reference Sample Set. Protein expression data for each sample in the Reference Sample Set were obtained for a panel of 1305 known proteins (SOMAscan 1.3k, SomaLogic, Boulder, CO, USA). Protein sets associated with relevant biological functions were established as the intersection of queries from GeneOntology and UniProt databases and the measured proteins. (These are listed in the Supplementary Materials.) Set enrichment methods used rank-based (Mann–Whitney) correlation of the 1305 measured proteins with test classifications of the reference samples, to examine correlation of test classifications with the selected biological functions. PSEA was implemented in C# following the approach of Subramanian et al. [49], as adapted by Roder et al. [50], for increased power, to detect associations and used *p*-values as defined therein. Briefly, the 1305 proteins were ranked based on the univariate correlation of their expression with test classification. An enrichment score was calculated which quantifies the enrichment in the rankings of the subset of the proteins related to a particular biological process relative to the proteins not related to that process. To increase the statistical power to identify associations between test classification and a biological process, the Reference Sample Set was divided into two halves multiple times; the enrichment score was evaluated for each half and then averaged over each half and each division. The *p*-values for association were determined by comparison with the null distribution generated via permutation of the test classifications over samples. False discovery rates were assessed by the method of Benjamini and Hochberg [51].

### 4.5. Statistics

Progression-free survival (PFS) was either measured from the time point of diagnosis (blood collection) to the date of disease recurrence/progression or death, or censored at last follow-up time, in the absence of either event. Overall survival (OS) was either measured from date of diagnosis to death, or censored at last follow-up time. All statistical analyses, apart from the PSEA, were performed by using SAS9.3 (SAS Institute, Cary, NC, USA) or PRISM (GraphPad, La Jolla, CA, USA). Survival plots and medians were created by using Kaplan–Meier methods. Difference in outcome between subgroups was assessed by using Cox proportional hazard *p*-values. The association between test classification and categorical or continuous variables was assessed by Fisher’s exact test and the Mann–Whitney test, respectively. The *p*-values were two-sided and uncorrected for multiple comparisons.

### 4.6. Data Availability

The Deep MALDI average spectra for all samples in this study are freely available at https://bitbucket.org/joannaroder/workspace/projects/OV.

## 5. Conclusions

This work highlights that, just like molecular markers of the tumor itself, the systemic condition of a patient (partly reflected in proteomic patterns) also influences survival and therapy response and could therefore be integrated into future processes of therapy planning.

## Figures and Tables

**Table 1:** Patient characteristics for development and validation cohort.

Characterstic		Development Cohort (*n* = 199) *n* (%)	Validation Cohort (*n* = 135) *n* (%)
Histology	serous	142 (71)	113 (84)
	non-serous	57 (29)	22 (16)
FIGO	1	36 (18)	0 (0)
	2	7(4)	8 (6)
	3	119 (60)	101 (75)
	4	37 (19)	26 (19)
Histologic	NA	2 (1)	0 (0)
Grade	1	11 (6)	4 (3)
	2	81 (41)	30 (22)
	3	105 (53)	101 (75)
Residual	yes	83 (42)	38 (28)
Tumor	no	113 (57)	97 (72)
	NA	3 (2)	0 (0)
Age	≤55	77 (39)	58 (43)
	>55	122 (61)	77 (57)
		Median (range)	Median (range)
Age		59 (18–88)	57 (27–85)

FIGO: Fédération Internationale de Gynécology et d’Obstétrique; NA: not available.

**Table 2:** Multivariate analysis of overall survival (OS) and progression-free survival (PFS) for younger patients in the DC and in the VC.

Development Cohort					
		OS		PFS	
		HR (95% CI)	*p*-Value	HR (95% CI)	*p*-Value
Test Classification (vs. poor)	good	0.23 (0.08–0.65)	0.005	0.31 (0.12–0.79)	0.014
FIGO (vs. IV)	I/II	0.24 (0.06–1.02)	0.054	0.12 (0.02–0.59)	0.009
	III	0.70 (0.29–1.72)	0.439	0.58 (0.25–1.36)	0.209
Histology (vs. serous)	non-serous	1.15 (0.45–2.91)	0.775	0.85 (0.31–2.33)	0.749
Grade (vs. 3)	1/2	0.76 (0.32–1.79)	0.533	1.35 (0.63–2.88)	0.440
Residual Tumor (vs. yes)	no	1.19 (0.39–3.62)	0.759	1.37 (0.48–3.87)	0.559
Validation Cohort					
		OS		PFS	
		HR (95% CI)	*p*-value	HR (95% CI)	*p*-value
Test Classification (vs. poor)	good	0.43 (0.16–1.20)	0.108	0.49 (0.23–1.06)	0.0686
FIGO (vs. IV)	I/II	0.14 (0.01–1.53)	0.108	0.08 (0.01–0.55)	0.010
	III	0.29 (0.09–0.92)	0.036	0.25 (0.08–0.78)	0.017
Histology (vs. serous)	non-serous	2.01 (0.61–6.60)	0.249	1.44 (0.57–3.62)	0.437
Grade (vs. 3)	1/2	0.72 (0.24–2.12)	0.548	0.49 (0.23–1.06)	0.070
Residual Tumor (vs. yes)	no	1.67 (0.63–4.43)	0.305	0.80 (0.26–2.44)	0.692

**Table 3:** Multivariate analysis, using the compound age/test classification attribute for the DC and the VC.

Development Cohort					
		OS		PFS	
		HR (95% CI)	*p*-Value	HR (95% CI)	*p*-Value
ProteomicAgeClassification		0.68 (0.56–0.83)	<0.001	0.85 (0.73–1.00)	0.047
FIGO (vs. IV)	I/II	0.17 (0.07–0.42)	<0.001	0.08 (0.03–0.21)	<0.001
	III	0.46 (0.28–0.75)	0.002	0.51 (0.30–0.84)	0.009
Histology (vs. serous)	non-serous	1.53 (0.94–2.48)	0.087	1.71 (1.04–2.81)	0.034
Grade (vs. 3)	1/2	0.97 (0.62–1.50)	0.881	1.35 (0.88–2.09)	0.171
Residual Tumor (vs. yes)	no	1.71 (1.06–2.77)	0.028	1.67 (1.05–2.64)	0.029
**Validation Cohort**					
		**OS**		**PFS**	
		**HR (95% CI)**	***p*-Value**	**HR (95% CI)**	***p*-Value**
ProteomicAgeClassification		0.81 (0.67–1.00)	0.045	0.83 (0.70–0.98)	0.030
FIGO (vs. IV)	I/II	0.62 (0.19–2.01)	0.428	0.26 (0.07–0.94)	0.040
	III	0.49 (0.28–0.84)	0.010	0.56 (0.32–0.96)	0.035
Histology (vs. serous)		0.88 (0.45–1.69)	0.695	0.79 (0.43–1.46)	0.458
Grade (vs. 3)	1/2	0.99 (0.56–1.76)	0.981	0.95 (0.58–1.54)	0.835
Residual Tumor (vs. yes)	no	2.20 (1.33–3.65)	0.002	1.74 (1.03–2.95)	0.039

## Supplementary Materials

The following are available online at https://www.mdpi.com/2072-6694/12/9/2519/s1, Figure S1: Performance of the tests trained using data from (i) patients aged ≤ 55 years or (ii) patients aged ≥ 56 years. Figure S2: Example of defined features. Figure S3: Classifier development approach. Figure S4: Process for the simultaneous refinement of training class labels and classifier. Table S1: Patient characteristics of the development cohort by test classification, good or poor. Table S2: Patient characteristics of the younger patients (age ≤ 55) in the development cohort by test classification, good or poor. Table S3: Patient characteristics of the older patients (age ≥ 56) in the development cohort by test classification, good or poor. Table S4: Patient characteristics by test classification for the validation cohort. Table S5: Additional univariate and multivariate analyses in the younger patients subgroup of the development cohort. Table S6: Additional multivariate analyses in the younger patients subgroup of the validation cohort. Table S7: Concordance table of test classifications between two runs of the 34 samples from the development cohort. Table S8: Concordance table of test classifications between plasma samples and serum samples for 37 patients in the development cohort. Table S9: PSEA assessment of association of test classifications with various biological processes. Table S10: Points in m/Z used to align the raster spectra. Table S11: Regions of spectrum used for first coarse normalization. Table S12: Regions of spectrum used for second normalization. Table S13: m/Z positions used for alignment. Table S14: m/Z regions (features) used for final normalization. Table S15: Definitions of features used in classifier development. References [52,53,54,55] are cited in the supplementary materials.
