# Supplementary material for: Definition and Independent Validation of a Proteomic-Classifier in Ovarian Cancer
Source: Cancers (Basel). 2020 Sep 4;12(9):2519. doi: 10.3390/cancers12092519 (PMC7564837; doi:10.3390/cancers12092519)
Supplement: Supplementary file 1 [file cancers-12-02519-s001.zip › cancers-873742- Supplementary Materials.pdf]

cancers

MDPI

*Article*

# Definition and Independent Validation of a Proteomic-Classifier in Ovarian Cancer

**Sabine Kasimir-Bauer, Joanna Roder, Eva Obermayr, Sven Mahner, Ignace Vergote, Liselore Loverix, Elena Braicu, Jalid Sehouli, Nicole Concin, Rainer Kimmig, Lelia Net, Heinrich Roder, Robert Zeillinger and Stefanie Aust**

## Supplementary Materials

## Supplementary Results

**Younger patient test applied to younger patient subgroup**

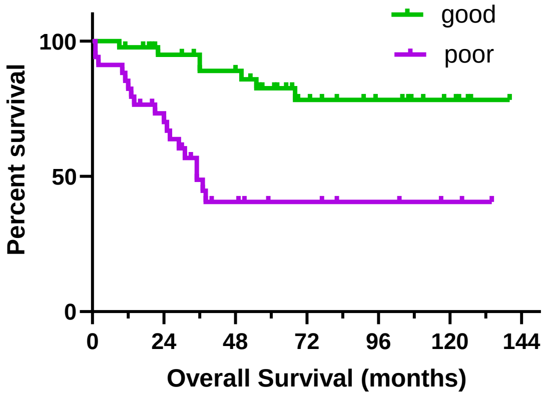


**Younger patient test applied to older patient subgroup**

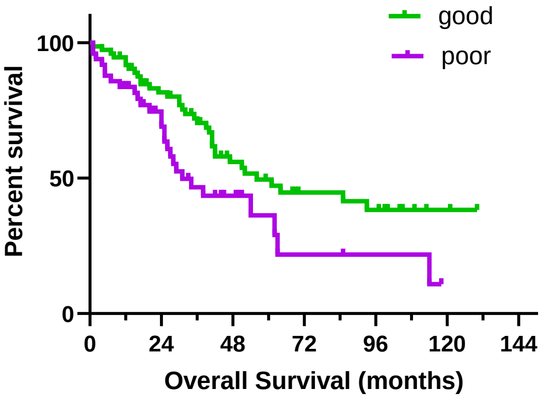


**Older patient test applied to younger patient subgroup**

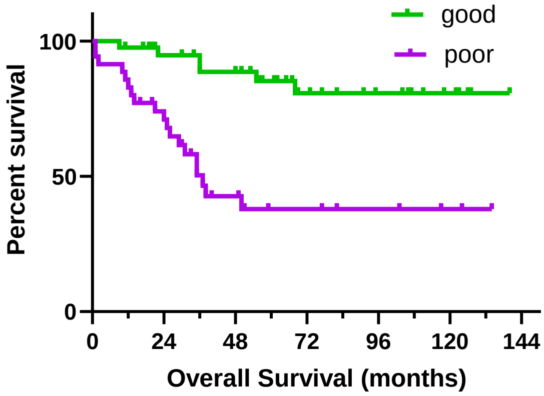


**Older patient test applied to older patient subgroup**

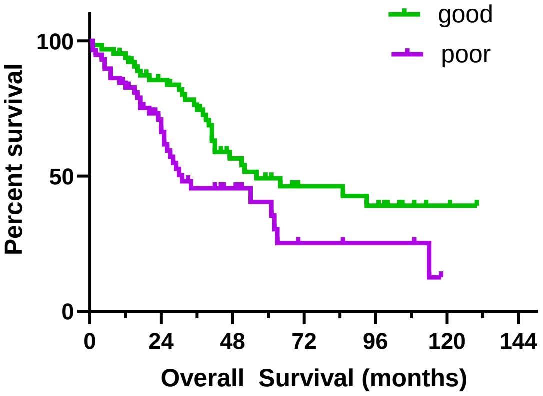


**Figure S1.** Performance of the tests trained using data from (i) patients aged ≤ 55 years or (ii) patients aged ≥ 56 years. Each test is applied to the subgroup of younger patients (≤ 55 years) and the subgroup of older patients (≥ 56 years). Kaplan-Meier plots of overall survival are shown.

**Table S1.** Patient characteristics of the development cohort by test classification, good or poor.

| | | Good (*N* = 106) | Poor (*N* = 93) | *p* value |
|---|---|---|---|---|
| | | Median (Range) | | |
| Age at diagnosis | | 59 (18–83) | 59 (29–88) | |
| | | *n* (%) | | |
| Histology | serous | 69 (65) | 73 (78) | 0.042 |
| | non-serous | 37 (35) | 20 (22) | |
| FIGO stage | IA | 13 (12) | 3 (3) | 0.002 * |
| | IB | 2 (2) | 0 (0) | |
| | IC | 15 (14) | 3 (3) | |
| | IIA | 0 (0) | 2 (2) | |
| | IIB | 2 (2) | 1 (1) | |
| | IIC | 0 (0) | 2 (2) | |
| | III | 5 (5) | 0 (0) | |
| | IIIA | 6 (6) | 1 (1) | |
| | IIIB | 9 (8) | 2 (2) | |
| | IIIC | 43 (41) | 53 (57) | |
| | IV | 11 (10) | 26 (28) | |
| Grade | 1 | 10 (9) | 1 (1) | 0.009 ** |
| | 2 | 46 (43) | 35 (38) | |
| | 3 | 48 (45) | 57 (61) | |
| | NA | 2 (2) | 0 (0) | |
| Residual Tumor Post Surgery | No | 78 (74) | 35 (38) | <0.001 *** |
| | Yes | 27 (25) | 56 (60) | |
| | NA | 1 (1) | 2 (2) | |

* stages I and II vs. stages III and IV; ** NA excluded; *** yes vs. no.

**Table S2.** Patient characteristics of the younger patients (age ≤ 55) in the development cohort by test classification, good or poor.

| | | Good (*N* = 42) | Poor (*N* = 35) | *p* value |
|---|---|---|---|---|
| | | Median (Range) | | |
| Age at diagnosis | | 43 (18–55) | 48 (29–55) | |
| | | *n* (%) | | |
| Histology | serous | 23 (55) | 27 (77) | 0.056 |
| | non-serous | 19 (45) | 8 (23) | |
| FIGO stage | IA | 8 (19) | 3 (9) | 0.019 * |
| | IB | 2 (5) | 0 (0) | |
| | IC | 10 (24) | 2(6) | |
| | IIA | 0 (0) | 1 (3) | |
| | IIB | 1 (2) | 1 (3) | |
| | IIC | 0 (0) | 1 (3) | |
| | III | 1 (2) | 0 (0) | |
| | IIIA | 1 (2) | 1 (3) | |
| | IIIB | 4 (10) | 1 (3) | |
| | IIIC | 10 (24) | 15 (43) | |
| | IV | 5 (12) | 10 (29) | |
| Grade | 1 | 6 (14) | 1 (3) | 0.120 ** |
| | 2 | 19 (45) | 15 (43) | |
| | 3 | 15 (36) | 19 (54) | |
| | NA | 2 (5) | 0 (0) | |
| Residual Tumor Post Surgery | No | 36 (86) | 13 (37) | <0.001 *** |
| | Yes | 5 (12) | 22 (63) | |
| | NA | 1 (2) | 0 (0) | |

* stages I and II vs. stages III and IV; ** NA excluded; *** yes vs. no.

**Table S3.** Patient characteristics of the older patients (age ≥ 56) in the development cohort by test classification, good or poor.

| | | **Good (*N* = 64)** | **Poor (*N* = 58)** | ***p* value** |
|---|---|---|---|---|
| | | Median (Range) | | |
| Age at diagnosis | | 65 (56–83) | 67 (56–88) | |
| | | *n* (%) | | |
| Histology | serous | 46 (72) | 46 (79) | 0.403 |
| | non-serous | 18 (28) | 12 (21) | |
| FIGO stage | IA | 5 (8) | 0 (0) | 0.048 * |
| | IB | 0 (0) | 0 (0) | |
| | IC | 5 (8) | 1 (2) | |
| | IIA | 0 (0) | 1 (2) | |
| | IIB | 1 (2) | 0 (0) | |
| | IIC | 0 (0) | 1 (2) | |
| | III | 4 (6) | 0 (0) | |
| | IIIA | 5 (8) | 0 (0) | |
| | IIIB | 5 (8) | 1 (2) | |
| | IIIC | 33 (52) | 38 (66) | |
| | IV | 6 (9) | 16 (28) | |
| Grade | 1 | 4 (6) | 0 (0) | 0.075 ** |
| | 2 | 27 (42) | 20 (34) | |
| | 3 | 33 (50) | 38 (66) | |
| | NA | 0 (0) | 0 (0) | |
| Residual Tumor Post Surgery | No | 42 (66) | 22 (38) | 0.006 *** |
| | Yes | 22 (34) | 34 (59) | |
| | NA | 0 (0) | 2 (3) | |

* stages I and II vs. stages III and IV; ** NA excluded *** yes vs. no.

**Table S4.** Patient characteristics by test classification for the validation cohort.

| | | **Good (*N* = 66)** | **Poor (*N* = 69)** | ***p* value** |
|---|---|---|---|---|
| | | Median (Range) | | |
| Age at diagnosis | | 62 (27–85) | 56 (35–81) | 0.140 |
| | | *n* (%) | | |
| Histology | serous | 56 (85) | 57 (83) | 0.817 |
| | non-serous | 10 (15) | 12 (17) | |
| FIGO stage | IIA | 2 (3) | 0 (0) | 0.159 * |
| | IIB | 1 (2) | 1 (1) | |
| | IIC | 3 (5) | 1 (0) | |
| | IIIA | 2 (3) | 0 (0) | |
| | IIIB | 2 (3) | 3 (4) | |
| | IIIC | 42 (64) | 52 (75) | |
| | IV | 14 (21) | 12 (17) | |
| Grade | 1 | 4 (6) | 0 (0) | 0.049 |
| | 2 | 17 (26) | 13 (19) | |
| | 3 | 45 (68) | 56 (81) | |
| Residual Tumor post surgery | No | 50 (76) | 47 (68) | 0.345 ** |
| | Yes | 16 (24) | 22 (32) | |
| | Yes: ≤ 0.5 cm | 5 | 3 | |
| | Yes: 0.5–1cm | 4 | 7 | |
| | Yes: 1–2cm | 2 | 3 | |
| | Yes: > 2 cm | 5 | 9 | |

* stages I and II vs. stages III and IV; ** yes vs. no.

**Table S5.** Additional Univariate and Multivariate Analyses in the Younger Patients Subgroup of the Development Cohort.

| | | OS | | PFS | |
|---|---|---|---|---|---|
| Univariate Analysis 1 | | HR (95% CI) | *p* value | HR (95% CI) | *p* value |
| Test Classification (vs. poor) | good | 0.18 (0.07–0.45) | <0.001 | 0.28 (0.14–0.58) | 0.001 |
| Univariate Analysis 2 | | HR (95% CI) | *p* value | HR (95% CI) | *p* value |
| Residual Tumor (vs. yes) | | 4.31 (1.90–9.74) | 0.001 | 5.43 (2.59–11.39) | <0.001 |
| Univariate Analysis 3 | | HR (95% CI) | *p* value | HR (95% CI) | *p* value |
| FIGO (vs. IV) | I/II | 0.17 (0.05–0.55) | 0.004 | 0.08 (0.02–0.27) | <0.001 |
| | III | 0.56 (0.23–1.36) | 0.203 | 0.51 (0.23–1.11) | 0.088 |
| Multivariate Analysis 1 | | HR (95% CI) | *p* value | HR (95% CI) | *p* value |
| Test Classification (vs. poor) | good | 0.26 (0.09–0.71) | 0.008 | 0.47 (0.21–1.08) | 0.074 |
| Residual Tumor (vs. yes) | no | 2.52 (1.04–6.11) | 0.042 | 3.79 (1.65–.8.69) | 0.002 |
| Multivariate Analysis 2 | | HR (95% CI) | *p* value | HR (95% CI) | *p* value |
| Test Classification (vs. poor) | good | 0.19 (0.07–0.50) | 0.001 | 0.30 (0.14–0.62) | 0.001 |
| FIGO (vs. IV) | I/II | 0.21 (0.06–0.72) | 0.013 | 0.08 (0.03–0.28) | <0.001 |
| | III | 0.70 (0.29–1.71) | 0.434 | 0.52 (0.24–1.13) | 0.096 |

**Table S6.** Additional Multivariate Analyses in the Younger Patients Subgroup of the Validation Cohort.

| | | OS | | PFS | |
|---|---|---|---|---|---|
| Univariate Analysis1 | | HR (95% CI) | *p* value | HR (95% CI) | *p* value |
| Test Classification (vs. poor) | good | 0.31 (0.12–0.79) | 0.015 | 0.48 (0.25–0.95) | 0.034 |
| Univariate Analysis 2 | | HR (95% CI) | *p* value | HR (95% CI) | *p* value |
| Residual Tumor (vs. yes) | | 3.02 (1.23–7.39) | 0.016 | 2.02 (0.88–4.63) | 0.098 |
| Univariate Analysis 3 | | HR (95% CI) | *p* value | HR (95% CI) | *p* value |
| FIGO (vs. IV) | I/II | 0.15 (0.02–1.19) | 0.073 | 0.12 (0.03–0.58) | 0.008 |
| | III | 0.24 (0.10–0.59) | 0.002 | 0.27 (0.12–0.60) | 0.001 |
| Multivariate Analysis 1 | | HR (95% CI) | *p* value | HR (95% CI) | *p* value |
| Test Classification (vs. poor) | good | 0.37 (0.14–0.99) | 0.047 | 0.52 (0.26–1.02) | 0.057 |
| Residual Tumor (vs. yes) | no | 2.16 (0.86–5.48) | 0.104 | 1.71 (0.73–4.00) | 0.214 |
| Multivariate Analysis 2 | | HR (95% CI) | *p* value | HR (95% CI) | *p* value |
| Test Classification (vs. poor) | good | 0.41 (0.15–1.10) | 0.076 | 0.60 (0.29–1.23) | 0.161 |
| FIGO (vs. IV) | I/II | 0.20 (0.03–1.70) | 0.142 | 0.15 (0.30–0.72) | 0.018 |
| | III | 0.34 (0.13–0.89) | 0.027 | 0.35 (0.15–0.83) | 0.017 |

Mean and median of each mass spectral feature by test classification together with univariate *t*-test *p* values and Mann-Whitney test *p* value are provided for each mass spectral feature used in the test in the spreadsheet UnivariateMSFeatureAssociation.xlsx.

**Table S7.** Concordance table of test classifications between two runs of the 34 samples from the development cohort.

| | | First Run | |
|---|---|---|---|
| | | Good ($n$ = 19) | Poor ($n$ = 15) |
| **Second Run** | Good ($n$ = 18) | 18 | 0 |
| | Poor ($n$ = 16) | 1 | 15 |

**Table S8.** Concordance table of test classifications between plasma samples and serum samples for 37 patients in the development cohort.

| | | Plasma | |
|---|---|---|---|
| | | Good | Poor |
| **Serum** | Good | 18 | 1 |
| | Poor | 3 | 15 |

Mann-Whitney test $p$ value and FDR for univariate association of each protein measured for the Reference Sample Set using the aptamer-based panel with test classification are provided in the spreadsheet ProteinPanelData.xlsx. This spreadsheet also shows which proteins are included in the subset associated with each biological process investigated.

**Table S9.** PSEA assessment of association of test classifications with various biological processes.

| Biological Process | $p$ Value of Association | FDR |
|---|---|---|
| Acute phase response | 0.0009 | <0.03 |
| Complement activation (narrowly defined) | 0.0023 | <0.03 |
| Acute inflammatory response | 0.0027 | <0.03 |
| Complement activation (broadly defined) | 0.0062 | <0.05 |
| EMT | 0.0527 | <0.30 |
| Angiogenesis | 0.0744 | <0.30 |
| Glycolysis | 0.0774 | <0.30 |
| Response to hypoxia | 0.1469 | <0.50 |
| Cellular component of morphogenesis | 0.2135 | <0.65 |
| Type 2 immune response | 0.3010 | <0.80 |
| Immune tolerance | 0.3631 | <0.85 |
| Immune tolerance and suppression | 0.3837 | <0.85 |
| NK cell mediated immunity | 0.4958 | <1.00 |
| Interferon Gamma | 0.5374 | <1.00 |
| Type 17 immune response | 0.5799 | <1.00 |
| Chronic inflammatory response | 0.6256 | <1.00 |
| Extracellular matrix organization | 0.6491 | <1.00 |
| Type 1 immune response | 0.6923 | <1.00 |
| Wound healing (narrowly defined) | 0.6925 | <1.00 |
| Wound healing (broadly defined) | 0.7352 | <1.00 |
| Cytokine production involved in immune response | 0.7536 | <1.00 |
| Behavior | 0.8221 | <1.00 |
| T cell mediated immunity | 0.8463 | <1.00 |
| Interferon type 1 | 0.8830 | <1.00 |
| Innate immune response | 0.9289 | <1.00 |
| B cell mediated immunity | 0.9542 | <1.00 |

## Supplementary Methods

## Detailed Description of Methods

### Sample Preparation

Samples were thawed. 3 μL aliquots were spotted onto serum cards (Therapak, Claremont, CA, USA) and allowed to dry for 1 h. A 6 mm skin biopsy punch (Acuderm, Fort Lauderdale, FL, USA) was used to cut out the entire dried plasma or serum spot. The punched out spot was added to 100 μ: of HPLC grade water (JT Baker, Phillipsburg, NJ, USA) in a centrifugal filter with a 0.45 μm nylon membrane (VWR, Radnor, PA, USA). The filter was vortexed gently for ten minutes and then spun at 14,000 rcf for two minutes. The filtrate was removed and transferred back to the punch for a second extraction cycle, which used three minutes of vortexing and spinning at 14,000 rcf again for two minutes. Twenty μL of the resulting filtrate was used for the MALDI analysis.

### Spectral Acquisition and Processing

*Acquisition*

MALDI spectra were acquired in positive ion mode on a MALDI-TOF mass spectrometer (SimulTOF 100 s/n: LinearBipolar 11.1024.01 from SimulTof Systems, Marlborough, MA, USA). This mass spectrometer uses a 349 nm, diode-pumped, frequency-tripled Nd:YLF laser. The laser was

operated at a repetition rate of 0.5 kHz. Prior to running experimental samples, an external calibration was performed using a mixture of standard proteins (Bruker Daltonics, Germany) consisting of insulin (*m*/*z* 5734.51 Da), ubiquitin (*m*/*z*, 8565.76 Da), cytochrome C (*m*/*z* 12360.97 Da), and myoglobin (*m*/*z* 16952.30 Da).

The position of the laser beam on the MALDI spot is controlled by moving the stage. This was carried out a rate of 0.25 mm/s. As the stage moves, the laser produces shots at different points on the MALDI sample spot. Spectra were collected, integrating the results from 800 laser shots at a time. Many such 800-shot spectra were collected; only spectra which did not meet a minimum intensity of 0.01 V were discarded.

**Processing of Raster Spectra to Deep MALDI Averages**

Many 800-shot spectra are averaged together to create a Deep MALDI spectrum for the sample. Full details of the Deep MALDI approach can be found in [14].

800-shot spectra were rescaled in the mass/charge (m/Z) axis relative to a standard reference spectrum to alignment on the m/Z axis. After a ripple filter was applied. Peaks in the spectra were detected on background-subtracted spectra. The resulting peak list was used for a second alignment step of the m/Z axis, to the alignment points listed in Table S10.

**Table S10.** Points in m/Z used to align the raster spectra.

| m/Z |
|---|
| 3168 |
| 4153 |
| 4183 |
| 4792 |
| 5773 |
| 5802 |
| 6433 |
| 6631 |
| 7202 |
| 7563 |
| 7614 |
| 7934 |
| 8034 |
| 8206 |
| 8684 |
| 8812 |
| 8919 |
| 8994 |
| 9133 |
| 9310 |
| 9427 |
| 10739 |
| 10938 |
| 11527 |
| 12173 |
| 12572 |
| 12864 |
| 13555 |
| 13763 |
| 13882 |
| 14040 |
| 14405 |
| 15127 |
| 15263 |
| 15869 |

| |
|---|
| 17253 |
| 18630 |
| 21066 |
| 23024 |
| 28090 |
| 28298 |

800-shot spectra with fewer than 20 detected peaks were discarded. In addition, if less than five of the points in Table S11 were used for alignment, a spectrum also failed quality control and was discarded.

The 400,000 laser shot Deep MALDI average spectrum for each sample was then created by averaging 500 randomly-selected 800-shot spectra. In the following, 'spectrum' will refer to the Deep MALDI average spectrum for a sample.

## Processing of Deep MALDI Average Spectra

Deep MALDI average spectra much be processed to make them reproducible and comparable between samples. The first step in this processing is subtraction of background, which is estimated out using the piecewise linear method of the convex hull. Spectra are then normalized using partial ion current (PIC) over the spectral regions defined in Table S11.

**Table S11.** Regions of spectrum used for first coarse normalization.

| m/Z Left | m/Z Right |
|---|---|
| 6100 | 7500 |
| 8500 | 10,700 |
| 13,300 | 15,000 |

Background subtraction and normalization are then repeated to better control spectral reproducibility. This time Eiler's method (Eiler's lambda = 4 and $p = 0.001$) is used for background estimation and the normalization uses the spectral regions defined in Table S12.

**Table S12.** Regions of spectrum used for second normalization.

| m/Z Left | m/Z Right |
|---|---|
| 6931.26 | 6963.03 |
| 6963.58 | 6978.64 |
| 6979.01 | 7011.52 |
| 7012.07 | 7030.07 |
| 7030.26 | 7039.99 |
| 7066.99 | 7084.26 |
| 13864.91 | 13924.11 |
| 13925.45 | 13959.08 |
| 13959.98 | 14002.58 |
| 14008.41 | 14076.57 |
| 14077.02 | 14122.31 |
| 14124.55 | 14179.70 |
| 14180.60 | 14228.58 |
| 14229.93 | 14279.70 |
| 14280.60 | 14323.20 |
| 14412.88 | 14457.73 |
| 14464.45 | 14514.23 |
| 14516.47 | 14570.28 |
| 14571.18 | 14618.26 |
| 21006.87 | 21124.88 |
| 21125.85 | 21221.13 |
| 21221.61 | 21322.70 |

A final alignment step was used to adjust peak position differences in the average spectra, using a set of 26 predetermined alignment points (Supplementary Table S13), known to work well on typical human serum samples

**Table S13.** m/Z positions used for alignment.

| m/Z |
|---|
| 3315.17 |
| 4153.33 |
| 4456.88 |
| 4709.91 |
| 5066.47 |
| 6432.85 |
| 6631.27 |
| 7934.36 |
| 8916.29 |
| 9423.10 |
| 9714.25 |
| 12868.19 |
| 13766.39 |
| 14044.69 |
| 14093.30 |
| 15131.43 |
| 15871.93 |
| 16077.64 |
| 17255.58 |
| 17383.45 |
| 18630.93 |
| 21069.05 |
| 21168.45 |
| 28084.44 |
| 28292.86 |
| 67150.37 |

Features were defined in the mass spectra. These were defined by visual inspection of an indication representative set of spectra. A feature is an m/Z region in the mass spectrum, specified by its lower m/Z limit and its upper m/Z limit. While features were defined based on the location of mass spectral peaks in typical spectra, for any individual spectrum the feature may or may not contain a well-defined mass spectral peak. An example of features defined is displayed in Figure S2 with reference spectra in blue and spectra from development set samples in red. Once the features were defined, they became parameters in the fully specified test. For each feature and spectrum, a feature value was defined as the integrated area under the spectrum within the feature. 277 features were defined.

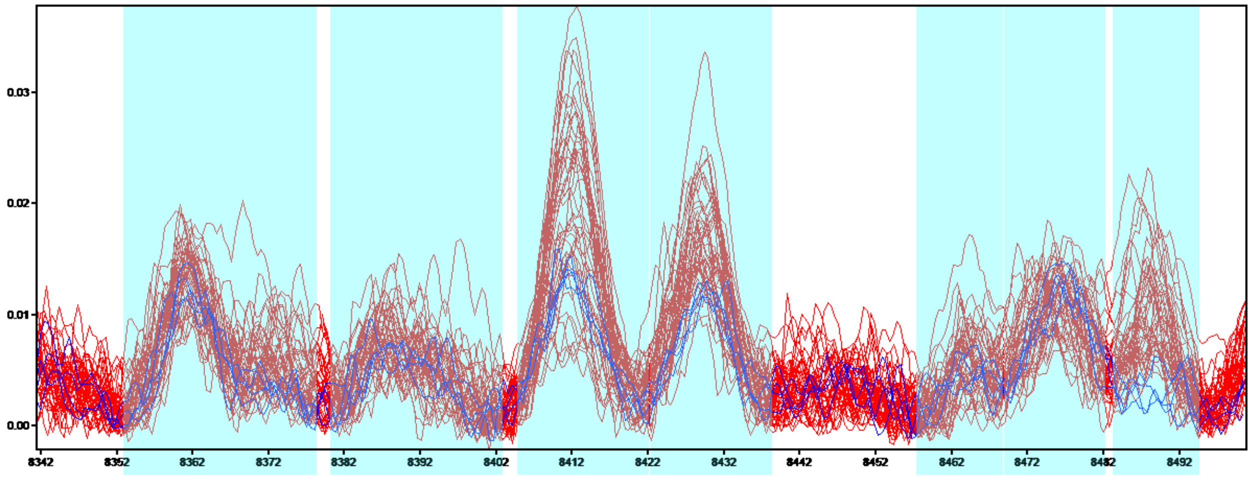


**Figure S2.** Example of defined features.

To ensure that spectral data can be reproducibly generated, the QC/reference samples that were included at the beginning and end of each batch of samples run were used to batch correct the feature values of each batch of samples. Batch correction parameters were determined by comparing the feature values of the QC/reference samples within the batch to gold standard values for the QC/reference sample defined from those contained within the first batch of the development set samples that were run. Note that development set samples and test samples were not used in determining batch correction parameters. Once the batch correction parameters were obtained for a particular batch, all feature values for each spectrum in the batch were corrected. This process adjusted for small m/Z dependent changes in mass spectral performance of the mass spectrometer or between mass spectrometers.

The final step in processing of the spectra was another PIC normalization step. Using the development set of spectra, a subset of the 277 defined features was defined for final spectral normalization that had low coefficients of variation and were not associated with any of the clinical outcome variables in the development set. The m/Z regions (also features) used and fixed as parameters in the fully-specified test are listed in Table S14.

**Table S14.** m/Z regions (features) used for final normalization.

| m/Z Left | m/Z Right |
|---|---|
| 3271.12 | 3295.67 |
| 3805.73 | 3829.9 |
| 4332.05 | 4351.22 |
| 4593.22 | 4605.96 |
| 4633.75 | 4651.09 |
| 6779.07 | 6817.17 |
| 6849.89 | 6868.99 |
| 7118.24 | 7169.66 |
| 7803.18 | 7837.46 |
| 8516.01 | 8541.91 |

| | |
|---|---|
| 10518.75 | 10610.01 |
| 14280.6 | 14323.2 |
| 20886.02 | 21427.39 |

The final normalization coefficient was calculated for each spectrum and all feature values were divided by the normalization coefficient for the spectrum/sample to produce the final processed feature values. Eight features were removed at this stage as, although they are useful in spectral processing, they are related to hemolysis and so ill-suited for use in classifier development. This left 269 features that were used for the development set samples for the creation of the classification algorithm. The definitions of these features are listed in Table S15.

**Table S15.** Definitions of features used in classifier development.

| m/Z Left | Center | m/Z Right |
|---|---|---|
| 3070.42 | 3082.6 | 3094.78 |
| 3095.15 | 3104.97 | 3114.79 |
| 3114.98 | 3129.7 | 3144.43 |
| 3144.81 | 3155.01 | 3165.2 |
| 3165.7 | 3177.13 | 3188.57 |
| 3188.61 | 3195.22 | 3201.83 |
| 3202.02 | 3213.35 | 3224.68 |
| 3227.7 | 3238.84 | 3249.98 |
| 3250.54 | 3260.27 | 3269.99 |
| 3271.12 | 3283.39 | 3295.67 |
| 3297.37 | 3315.78 | 3334.18 |
| 3346.46 | 3361.09 | 3375.72 |
| 3384.12 | 3392.9 | 3401.68 |
| 3404.89 | 3419.05 | 3433.21 |
| 3434.51 | 3443.9 | 3453.3 |
| 3453.79 | 3461.35 | 3468.9 |
| 3540.72 | 3555.53 | 3570.35 |
| 3583.14 | 3593.09 | 3603.05 |
| 3665.83 | 3678.85 | 3691.88 |
| 3692.83 | 3701.7 | 3710.57 |
| 3761.55 | 3772.5 | 3783.45 |
| 3805.73 | 3817.82 | 3829.9 |
| 3830.28 | 3839.91 | 3849.54 |
| 3883.43 | 3890.51 | 3897.59 |
| 3897.78 | 3905.52 | 3913.26 |
| 3913.45 | 3926.1 | 3938.75 |
| 3939.13 | 3949.89 | 3960.65 |
| 3999.11 | 4011.41 | 4023.71 |
| 4024.28 | 4030.32 | 4036.36 |
| 4036.93 | 4048.54 | 4060.15 |
| 4080.14 | 4094.83 | 4109.53 |
| 4112.17 | 4119.37 | 4126.57 |
| 4127.25 | 4133.39 | 4139.52 |
| 4198.95 | 4210.81 | 4222.68 |
| 4241.73 | 4249.86 | 4258 |
| 4258.83 | 4266.5 | 4274.18 |
| 4276.79 | 4289.24 | 4301.69 |
| 4332.05 | 4341.63 | 4351.22 |
| 4351.4 | 4359.83 | 4368.27 |
| 4372.4 | 4381.03 | 4389.66 |
| 4397.29 | 4407.28 | 4417.26 |
| 4427.4 | 4433.22 | 4439.04 |
| 4439.75 | 4443.96 | 4448.18 |
| 4449.38 | 4461.23 | 4473.07 |

| | | |
|---|---|---|
| 4502.53 | 4508.75 | 4514.98 |
| 4553.3 | 4565.57 | 4577.84 |
| 4580.87 | 4586.84 | 4592.81 |
| 4593.22 | 4599.59 | 4605.96 |
| 4618.51 | 4625.99 | 4633.46 |
| 4633.75 | 4642.42 | 4651.09 |
| 4667.54 | 4679.99 | 4692.43 |
| 4698.76 | 4713.31 | 4727.86 |
| 4747.49 | 4755.82 | 4764.15 |
| 4770.57 | 4776.34 | 4782.12 |
| 4782.62 | 4790.85 | 4799.08 |
| 4807.16 | 4819.05 | 4830.95 |
| 4845.9 | 4857.7 | 4869.49 |
| 4885.05 | 4893.33 | 4901.61 |
| 4910.19 | 4919.12 | 4928.06 |
| 4928.26 | 4938.24 | 4948.23 |
| 4949.44 | 4964.3 | 4979.15 |
| 4989.38 | 5000.07 | 5010.76 |
| 5012.17 | 5020.4 | 5028.63 |
| 5033.64 | 5041.17 | 5048.71 |
| 5048.95 | 5054.98 | 5061 |
| 5061.1 | 5070.88 | 5080.67 |
| 5093.87 | 5106.47 | 5119.06 |
| 5162.99 | 5185.8 | 5208.61 |
| 5209.58 | 5224.44 | 5239.3 |
| 5274.04 | 5288.09 | 5302.14 |
| 5351.59 | 5362.36 | 5373.12 |
| 5398.65 | 5408.14 | 5417.63 |
| 5424.89 | 5431.54 | 5438.18 |
| 5440.4 | 5452.78 | 5465.16 |
| 5512.62 | 5520.5 | 5528.38 |
| 5540.44 | 5552.25 | 5564.06 |
| 5564.15 | 5573.62 | 5583.09 |
| 5685.16 | 5693.39 | 5701.62 |
| 5701.82 | 5708.3 | 5714.77 |
| 5714.97 | 5720.49 | 5726.01 |
| 5726.03 | 5734.42 | 5742.81 |
| 5743.56 | 5750.24 | 5756.93 |
| 5757.29 | 5764.16 | 5771.03 |
| 5771.12 | 5778.62 | 5786.11 |
| 5786.29 | 5794.96 | 5803.62 |
| 5803.89 | 5810.08 | 5816.27 |
| 5816.42 | 5822.76 | 5829.11 |
| 5832.02 | 5840.46 | 5848.89 |
| 5850.08 | 5863.91 | 5877.73 |
| 5879.59 | 5888.74 | 5897.9 |
| 5898.07 | 5909.77 | 5921.47 |
| 5922.62 | 5934.73 | 5946.84 |
| 5949.41 | 5963.9 | 5978.4 |
| 5978.83 | 5987.76 | 5996.69 |
| 5998.01 | 6008.58 | 6019.14 |
| 6020.13 | 6028.93 | 6037.72 |
| 6054.61 | 6061.94 | 6069.27 |
| 6069.47 | 6082.86 | 6096.26 |
| 6099.57 | 6109.12 | 6118.68 |
| 6134.48 | 6148.68 | 6162.88 |
| 6165.75 | 6175.04 | 6184.34 |
| 6186.65 | 6194.45 | 6202.25 |

| | | |
|---|---|---|
| 6202.51 | 6214.44 | 6226.37 |
| 6275.16 | 6284.15 | 6293.14 |
| 6293.16 | 6301.49 | 6309.82 |
| 6322.27 | 6331.46 | 6340.64 |
| 6378.77 | 6393.09 | 6407.42 |
| 6409.41 | 6479.04 | 6548.68 |
| 6553.89 | 6564.68 | 6575.47 |
| 6575.85 | 6589.26 | 6602.67 |
| 6604.74 | 6675.06 | 6745.39 |
| 6779.07 | 6798.12 | 6817.17 |
| 6825.83 | 6837.67 | 6849.52 |
| 6849.89 | 6859.44 | 6868.99 |
| 6889.03 | 6896.99 | 6904.95 |
| 6930.88 | 6939.55 | 6948.22 |
| 6948.87 | 6956.18 | 6963.49 |
| 6963.58 | 6971.11 | 6978.64 |
| 6979.01 | 6995.27 | 7011.52 |
| 7011.77 | 7019.83 | 7027.88 |
| 7029.37 | 7033.6 | 7037.84 |
| 7037.91 | 7046.82 | 7055.73 |
| 7055.81 | 7060.15 | 7064.5 |
| 7065.49 | 7072.9 | 7080.31 |
| 7118.24 | 7143.95 | 7169.66 |
| 7178.66 | 7189.32 | 7199.97 |
| 7234.04 | 7243.67 | 7253.3 |
| 7279.59 | 7292.85 | 7306.11 |
| 7309.51 | 7318.12 | 7326.73 |
| 7327.41 | 7332.74 | 7338.06 |
| 7375.19 | 7390.07 | 7404.95 |
| 7406.19 | 7448.51 | 7490.84 |
| 7729.12 | 7735.87 | 7742.63 |
| 7742.75 | 7751.34 | 7759.93 |
| 7760.24 | 7767.77 | 7775.3 |
| 7776.52 | 7788.92 | 7801.31 |
| 7803.18 | 7820.32 | 7837.46 |
| 7984.8 | 7994.91 | 8005.01 |
| 8006.66 | 8018.69 | 8030.72 |
| 8131.01 | 8153.05 | 8175.09 |
| 8192.54 | 8215.68 | 8238.82 |
| 8306.66 | 8314.7 | 8322.74 |
| 8353.19 | 8366.02 | 8378.85 |
| 8401.71 | 8411.17 | 8420.63 |
| 8420.71 | 8428.79 | 8436.87 |
| 8466.84 | 8474.84 | 8482.84 |
| 8483.32 | 8489.05 | 8494.77 |
| 8516.01 | 8528.96 | 8541.91 |
| 8555.29 | 8565.12 | 8574.94 |
| 8575.31 | 8592.03 | 8608.74 |
| 8650.35 | 8659.11 | 8667.86 |
| 8754.04 | 8766.76 | 8779.48 |
| 8799.09 | 8820.53 | 8841.97 |
| 8860.56 | 8871.76 | 8882.96 |
| 8882.98 | 8891.91 | 8900.84 |
| 8904.09 | 8925.16 | 8946.24 |
| 8954.36 | 8961.34 | 8968.33 |
| 8968.81 | 8978.23 | 8987.65 |
| 8988.02 | 8998.68 | 9009.33 |
| 9010.43 | 9019.53 | 9028.62 |

| | | |
|---|---|---|
| 9028.78 | 9037.31 | 9045.84 |
| 9066.55 | 9077.91 | 9089.26 |
| 9089.32 | 9096.91 | 9104.51 |
| 9112.47 | 9133.46 | 9154.45 |
| 9196.31 | 9207.88 | 9219.45 |
| 9234.27 | 9243.94 | 9253.6 |
| 9254.17 | 9263.3 | 9272.44 |
| 9272.68 | 9289.41 | 9306.14 |
| 9308.35 | 9319.83 | 9331.31 |
| 9341.1 | 9374.82 | 9408.53 |
| 9411.21 | 9454.03 | 9496.84 |
| 9510.87 | 9520.27 | 9529.67 |
| 9560.23 | 9585.25 | 9610.26 |
| 9613.48 | 9626.56 | 9639.65 |
| 9640.56 | 9654.2 | 9667.85 |
| 9688.55 | 9723.57 | 9758.58 |
| 9903.45 | 9934.33 | 9965.21 |
| 10128.04 | 10139.87 | 10151.71 |
| 10152.46 | 10161.84 | 10171.22 |
| 10171.98 | 10184.57 | 10197.16 |
| 10197.54 | 10211.07 | 10224.6 |
| 10249.52 | 10262.23 | 10274.94 |
| 10295.62 | 10305.69 | 10315.75 |
| 10328.34 | 10350.14 | 10371.93 |
| 10435.64 | 10450.45 | 10465.26 |
| 10465.61 | 10482.62 | 10499.63 |
| 10518.75 | 10564.38 | 10610.01 |
| 10615.18 | 10638.37 | 10661.56 |
| 10711.79 | 10737.82 | 10763.85 |
| 10769.76 | 10776.18 | 10782.59 |
| 10782.96 | 10791.09 | 10799.22 |
| 10828.47 | 10847.99 | 10867.5 |
| 10951.44 | 10963.37 | 10975.3 |
| 11028.77 | 11056.4 | 11084.03 |
| 11090.89 | 11107.43 | 11123.96 |
| 11132.45 | 11152.43 | 11172.4 |
| 11285.82 | 11305.1 | 11324.39 |
| 11378.42 | 11392.26 | 11406.11 |
| 11428.16 | 11442.74 | 11457.32 |
| 11468.24 | 11485.3 | 11502.35 |
| 11513.71 | 11530.99 | 11548.26 |
| 11567.26 | 11584.42 | 11601.59 |
| 11611.34 | 11634.82 | 11658.3 |
| 11670.69 | 11686.46 | 11702.22 |
| 11719.74 | 11732.72 | 11745.69 |
| 11746.38 | 11756.13 | 11765.89 |
| 11769.8 | 11786.1 | 11802.4 |
| 11826.75 | 11843.48 | 11860.2 |
| 11876.81 | 11889.88 | 11902.95 |
| 11903.39 | 11913.25 | 11923.11 |
| 11927.82 | 11938.26 | 11948.69 |
| 11974.12 | 11997.34 | 12020.56 |
| 12084.48 | 12116.9 | 12149.32 |
| 12151.24 | 12160.63 | 12170.03 |
| 12266.86 | 12290.16 | 12313.47 |
| 12549.12 | 12574.49 | 12599.85 |
| 12645.37 | 12676.19 | 12707.01 |
| 12723.06 | 12738.33 | 12753.59 |

| | | |
|---|---|---|
| 12769.89 | 12789.06 | 12808.24 |
| 12834.49 | 12917.52 | 13000.55 |
| 13018.32 | 13031.4 | 13044.48 |
| 13049.54 | 13076.86 | 13104.18 |
| 13119.56 | 13135.29 | 13151.02 |
| 13265.3 | 13276.12 | 13286.94 |
| 13304.84 | 13325.96 | 13347.09 |
| 13351.99 | 13364.15 | 13376.31 |
| 13501.19 | 13524.33 | 13547.48 |
| 13554.22 | 13569.52 | 13584.82 |
| 13602.38 | 13612.58 | 13622.78 |
| 13708.2 | 13723.6 | 13739 |
| 13783.92 | 13795.98 | 13808.04 |
| 13860.73 | 13881.13 | 13901.52 |
| 13905.76 | 13917.74 | 13929.71 |
| 13929.96 | 13944.37 | 13958.78 |
| 13959.98 | 13981.28 | 14002.58 |
| 14014.11 | 14067.59 | 14121.06 |
| 14122.86 | 14174.53 | 14226.2 |
| 14229.93 | 14254.82 | 14279.7 |
| 14280.6 | 14301.9 | 14323.2 |
| 14401.51 | 14431.22 | 14460.94 |
| 14462.27 | 14541.41 | 14620.56 |
| 14623.06 | 14642.87 | 14662.69 |
| 14684.56 | 14699.66 | 14714.76 |
| 14764.89 | 14786.87 | 14808.84 |
| 14859.96 | 14882.15 | 14904.35 |
| 18248.47 | 18271.03 | 18293.59 |
| 18548.49 | 18570.16 | 18591.84 |
| 18603.02 | 18630.68 | 18658.34 |
| 18708.84 | 18730.03 | 18751.21 |
| 18811.43 | 18848.65 | 18885.87 |
| 20739.17 | 20758.97 | 20778.76 |
| 20886.02 | 21156.71 | 21427.39 |
| 21669.84 | 21804.69 | 21939.55 |
| 22566.7 | 22604.25 | 22641.79 |
| 22999.81 | 23033.14 | 23066.47 |
| 23097.51 | 23130.26 | 23163.02 |
| 23213.01 | 23246.92 | 23280.82 |
| 23305.86 | 23353.04 | 23400.22 |
| 23429.2 | 23467.05 | 23504.91 |
| 25144.7 | 25185.27 | 25225.84 |
| 25429.35 | 25473.61 | 25517.87 |
| 25519.61 | 25570.37 | 25621.14 |
| 25624.4 | 25686.01 | 25747.63 |
| 27915.48 | 27962.78 | 28010.08 |
| 28037.85 | 28133.53 | 28229.2 |
| 28237.01 | 28338.55 | 28440.09 |
| 28800.67 | 28859.9 | 28919.13 |
| 28924.34 | 28972.72 | 29021.1 |
| 29030.65 | 29078.6 | 29126.55 |

Note that while feature definitions are defined to two decimal places, peaks are not resolved to this accuracy. Mass resolution (m/Z location of peak/peak width at half height) is 343 at 3 kDa and 284 at 28 kDa.

## Development of the Classification Algorithm

### 1. A Hierarchical Classifier Development Platform Designed for Problems Where the Number of Available Instances is Smaller than the Number of Measured Attributes

The method of classifier development used is outlined in Figure S3. This platform has been used in several other personalized medicine projects (e.g., [34,35,52] and a detailed description can be found in [15]. It incorporates aspects of traditional and modern machine learning, including bagging, boosting, and regularization using dropout, with the aim of producing classifiers with reliable performance estimates from relatively small sample sets while minimizing chances of overfitting to peculiarities in the development set data.

The platform structure is illustrated schematically in Figure S3.

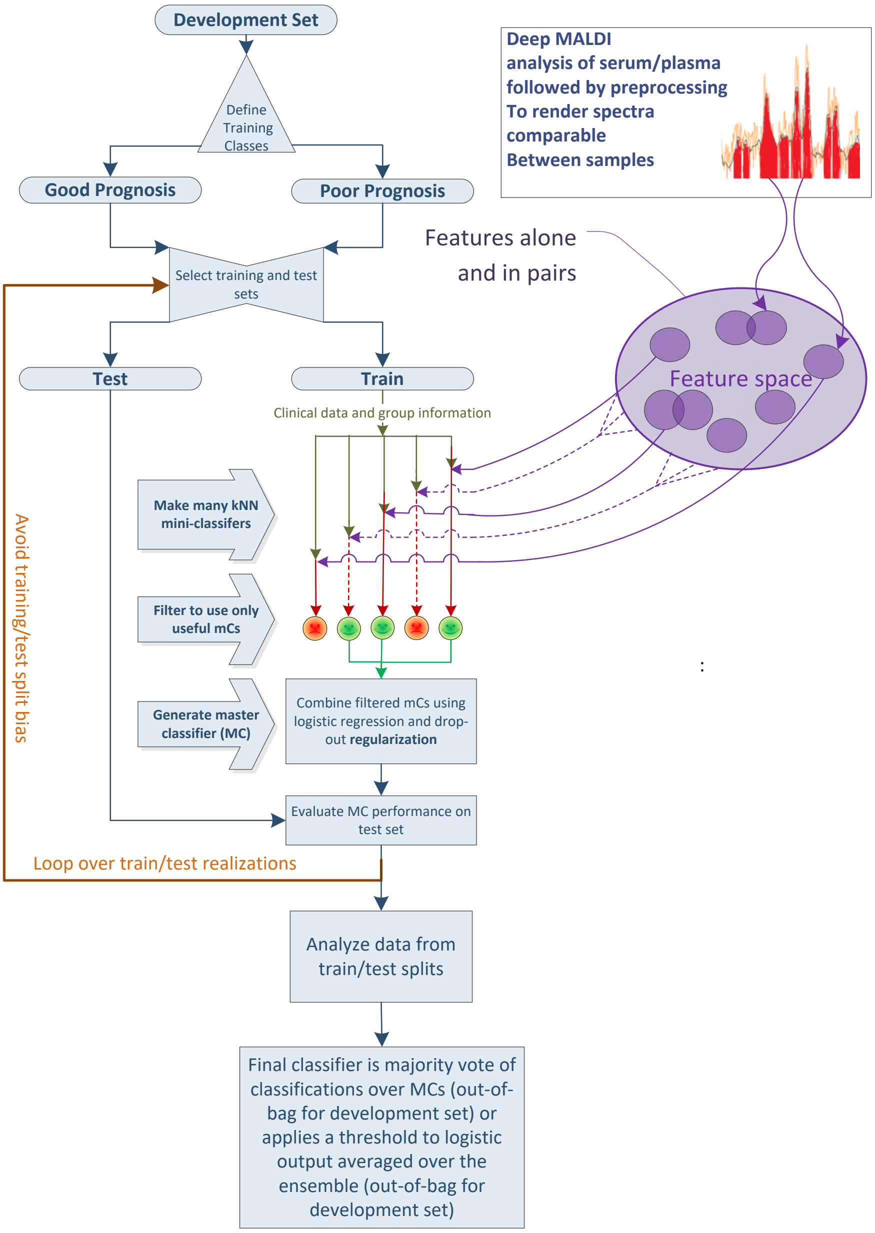


**Figure S3.** Classifier Development Approach.

The set of samples available for development was randomly split into training and test sets many times. This prevented the use of one training/test split that may be particularly easy or hard to classify in testing or particularly poor for training. An eventual ensemble average ("bagging"[53]) over these training/test split realizations allowed every sample in the development set to contribute to the

performance estimate of the final classifier via an "out-of-bag" estimate [18], i.e., the classification for a given sample in the development set was evaluated only over the subset of realizations where the sample is in the test set and not in the training set. This allowed for more reliable and generalizable classifier performance estimates to be generated from the development set alone.

For each training/test split realization, many classifiers ("atomic classifiers") were built using subsets of the mass spectral features. Here we used k-nearest neighbor classifiers with fixed k = 9 for atomic classifiers and used all single features and all possible combinations of pairs of features. No optimization was carried out for different values of k. We have found in previous work that typically good performance is achieved when k is chosen to be of the order of $\sqrt{\# \, samples}$ . Each atomic classifier was applied to its training set and the performance of the resulting classification groups assessed in terms of hazard ratio for overall survival between classification groups. The atomic classifiers were filtered so that only classifiers demonstrating acceptable performance (i.e., where the hazard ratio achieved on the training set between classification groups exceeded a minimal level) were used further in the platform. The performance of the individual atomic classifiers did not need to be excellent, as they were later combined. This approach uses the idea of boosting [54]—that many classifiers of decent performance can be combined into an overall classifier with at least as good, or better, performance. The use of many atomic classifiers increases test robustness, again adding more protection against overfitting, as we were not selecting the few top performing classifiers or features based on their apparently superior performance on the training set.

Once the atomic classifiers were filtered and poorly performing classifiers eliminated, the remaining atomic classifiers were combined to create one base classifier per training/test split realization. This was done using logistic regression over the training set samples. As there were very many atomic classifiers that pass filtering, it is essential to employ a strong regularizer to avoid overfitting during the regression. We used the concept of dropout, a technique commonly used as the regularizer during the training of deep learning nets [55] Our regularization method was implemented as follows. From the pool of atomic classifiers passing filtering, we randomly selected 10 atomic classifiers. We performed the logistic regression to calculate weights for combining this subset of atomic classifiers. We repeated this many times, enough that each atomic classifier was drawn many times, each dropout iteration drawing a random set of 10 atomic classifiers. The weight for each atomic classifier was averaged over many dropout iterations to give the weights for the final logistic combination. The continuous variable output of the logistic combination was converted to a single binary output by applying a threshold of 0.5.

The final level of the platform hierarchy was an ensemble average of the base classifiers (bagging over the training/test split realizations). This was carried out as a majority vote of binary outputs. In order to obtain meaningful performance estimates from the development set of samples, it was necessary to adapt the majority vote to an out-of-bag estimate, in which the majority vote for a particular sample was carried out only over the subset of base classifiers for which the sample was not included in the training set.

## 2. Training Class Definition and a Semi-Supervised Approach to Simultaneous Refinement of Training Class Labels and Classifier

This approach used supervised learning, i.e., it was necessary to know the training class labels for the classification problem, in this instance, which samples were from patients in the group likely to have good prognosis and which were not. It was not a priori clear how to unambiguously define good and poor prognosis in a way that revealed underlying information in the molecular data. We employed an approach that simultaneously refined training class labels for classifier development at the same time as the classifier itself [16]. This is shown schematically in Figure S4.

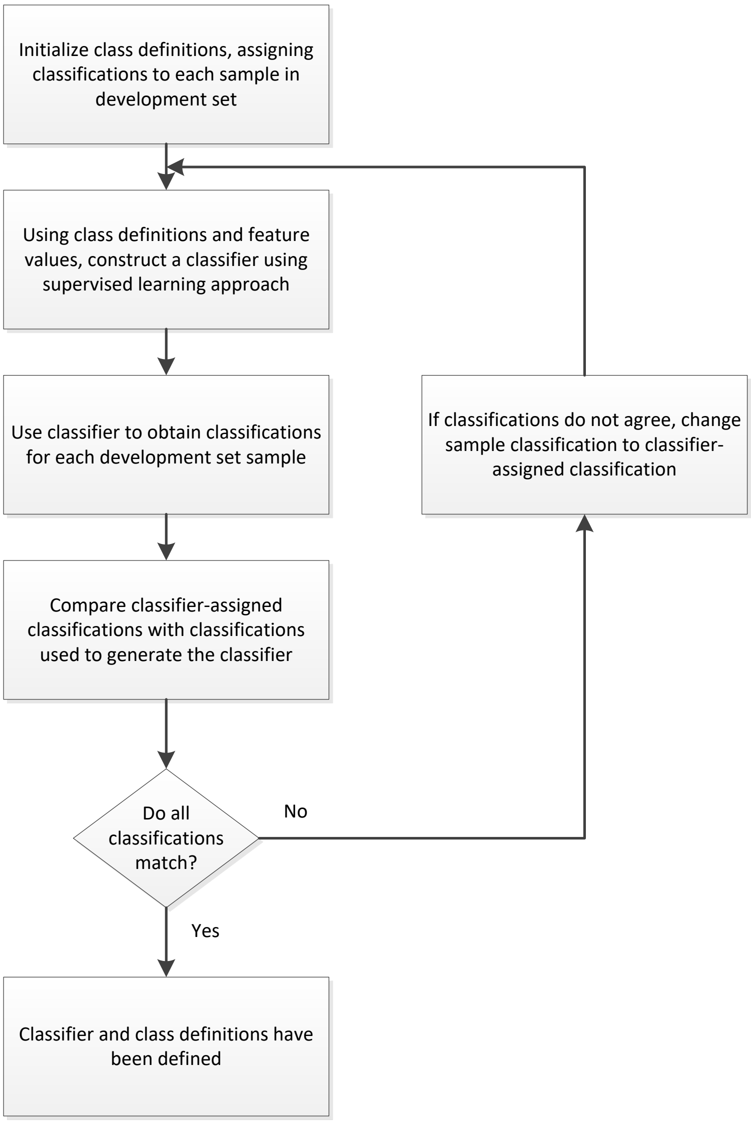


**Figure S4.** Process for the simultaneous refinement of training class labels and classifier.

First an educated guess was made for training class labels. The samples were sorted according to patient survival and the patients with the longest survival were assigned to the good prognosis group and the others to the poor prognosis group. Using these class labels, a classifier was constructed using the hierarchical approach explained above. Once the classifier was created, it was used to classify the samples in the development set or subset, with reliable and unbiased classifications obtained using its 'out-of-bag' approach. These classifications were used as the training class labels to create a second classifier, which reclassified the development subset samples, producing an iterative process. This process converged after four iterations. The result was a classifier together with a consistent set of training labels for the development set.

For this project we carried out the process described above using either data from the younger patients or data from the older patients. The performance of the resulting classifiers was quite similar, and we chose to use the classifier trained using only data from the older patients for all further investigations. All results shown for the older patients in the development set are out-of-bag estimates [18], to prevent over-estimation of classifier performance. All results shown for the younger patients apply the full classifier directly, as data from the younger patients was not used in classifier training.
